# Nucleotide Excision Repair in *Caenorhabditis elegans*

**DOI:** 10.4061/2011/542795

**Published:** 2011-08-17

**Authors:** Hannes Lans, Wim Vermeulen

**Affiliations:** Department of Genetics, Medical Genetics Center, Erasmus MC, P.O. Box 2040, 3000 CA Rotterdam, The Netherlands

## Abstract

Nucleotide excision repair (NER) plays an essential role in many organisms across life domains to preserve and faithfully transmit DNA to the next generation. In humans, NER is essential to prevent DNA damage-induced mutation accumulation and cell death leading to cancer and aging. NER is a versatile DNA repair pathway that repairs many types of DNA damage which distort the DNA helix, such as those induced by solar UV light. A detailed molecular model of the NER pathway has emerged from *in vitro* and live cell experiments, particularly using model systems such as bacteria, yeast, and mammalian cell cultures. In recent years, the versatility of the nematode *C. elegans* to study DNA damage response (DDR) mechanisms including NER has become increasingly clear. In particular, *C. elegans* seems to be a convenient tool to study NER during the UV response *in vivo*, to analyze this process in the context of a developing and multicellular organism, and to perform genetic screening. Here, we will discuss current knowledge gained from the use of *C. elegans* to study NER and the response to UV-induced DNA damage.

## 1. DNA Damage Response Mechanisms

To preserve and faithfully transmit DNA to the next generation, cells are equipped with a variety of DNA repair pathways and associated DNA damage responses, collectively referred to as the DNA damage response (DDR). DNA is continuously damaged by environmental and metabolism-derived genotoxic agents. It is vital for cells and organisms to properly cope with DNA damage, because unrepaired damage can give rise to mutation and cell death. The importance of the DDR is illustrated by several human cancer prone and/or progeroid hereditary diseases, which are based on defects in the DDR. Over the last decades, a wealth of information on the molecular mechanism of different repair pathways has been gathered from detailed *in vitro *and live cell studies. Currently, this acquired knowledge is being used to develop therapeutic strategies to treat patients suffering from the consequences of unrepaired DNA damage, such as cancer and aging [1]. 

**Figure 1:**
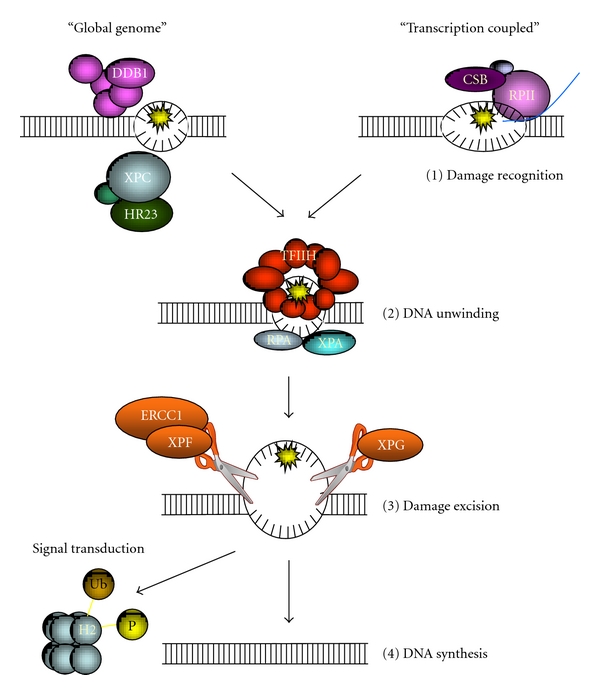
NER mechanism. DNA damage removal by NER is roughly executed in four subsequent steps. First, damage is recognised during transcription by stalling of RNA polymerase and involving CSB (“transcription coupled”), or it is recognised throughout the rest of the genome by the UV-DDB and XPC/HR23B complexes (“global genome”). Upon recognition, the TFIIH complex is recruited to unwind DNA around the damage and structural proteins XPA and RPA bind the resulting single-stranded DNA. Next, endonucleases ERCC1/XPF and XPG excise a patch of DNA including the damage. Finally, gap filling by *de novo *DNA synthesis takes place. During processing of a lesions, other proteins in proximity, including histones, are modified as part of a signalling cascade.

Damage is repaired by different DNA repair pathways depending on the type of DNA lesion, genomic location, and the cell cycle phase (for reviews see [2–4]). Lesions originating from different genotoxic sources can range from small base modifications to double-strand breaks. Small base modifications, such as oxidative lesions which do not substantially distort the double helix, are repaired by base excision repair (BER). BER removes single or several bases and repairs the gap by DNA synthesis. Bigger lesions to one strand of the DNA which substantially distort the DNA helix are repaired by nucleotide excision repair (NER). NER repairs lesions by cutting out a patch of the damaged DNA strand and filling in the gap by DNA synthesis (see below and Figure 1). More rigid lesions, which covalently crosslink both strands of the DNA, are repaired by interstrand crosslink (ICL) repair. Its precise repair mechanism is still poorly understood, but it involves several unique proteins of the Fanconi Anemia pathway and proteins that function in other repair pathways as well. Finally, DNA double-strand breaks (DSBs) are repaired by either homologous recombination (HR) or nonhomologous end-joining (NHEJ) or other alternative DSB repair pathways. HR is an error-free repair pathway and utilizes a homologous chromosome or sister chromatid, which is only present in late S- or G2-phase of the cell cycle, to repair damage. NHEJ is capable of rejoining broken DNA ends also in G1-phase and noncycling cells. However, due to processing of DNA ends prior to ligation, NHEJ is more error prone than HR. Although distinct classes of lesions are repaired by specific repair pathways, these pathways may compete for specific lesions or on the contrary, share common factors. In addition, several repair factors display multiple functions in DNA metabolism such as replication and transcription. These features show that the different repair pathways and other cellular responses to DNA damage form an interwoven intricate network. To fully understand DDR, it is, therefore, not sufficient to study a single repair process in isolation.

Much of the available knowledge regarding DDR mechanisms has come through the use of different model systems, such as bacteria, yeast, and cultured mammalian cells, and to a lesser extent of whole mice. The nematode *C. elegans* is increasingly being used to study various biological processes, including DNA repair [5–7]. This paper focuses on the function of NER in *C. elegans *and on the central role of this pathway in the cellular response to UV-induced DNA damage. 

## 2. Nucleotide Excision Repair

Many organisms are continuously exposed to solar UV irradiation. Although the vast majority of UV light emitted by the sun is blocked by the earth's ozone layer, penetrating UV light can still severely damage DNA directly and is thought to be a major cause of skin cancer in humans [8]. UV radiation can cause a range of different DNA lesions of which cyclobutane pyrimidine dimers (CPDs) and 6-4 photoproducts (64PPs) are most abundant [9]. CPD and 64PP lesions distort the double helix structure of DNA, thereby severely impeding vital processes such as transcription and replication. If these lesions are not repaired properly, error-prone replication can induce mutations leading to cancer or cause cells to die, which contributes to aging [10]. Although predominantly skin cells of bigger organisms are exposed to solar UV irradiation, many other agents, such as chemotherapeutics, cigarette smoke, toxins, and some food-contained chemicals, can cause similar helix distortions in other cells. Therefore, it is of major importance that cells are equipped with a mechanism to deal with this type of DNA damage. In bacteria, eukarya, and probably also in archaea, nucleotide excision repair (NER) functions to remove this wide range of helix-distorting lesions. 

In the budding yeast *Saccharomyces cerevisiae* and in mammals, in which NER has been extensively studied, NER is executed in roughly four subsequent steps: (1) lesion detection, (2) local unwinding and damage verification, (3) incision of the DNA surrounding the lesion, and finally (4) DNA synthesis and ligation to fill the resulting gap (Figure 1; for reviews, see [11, 12]). DNA damage that occurs in the active strand of transcribed genes is repaired by transcription-coupled NER (TC-NER). TC-NER is thought to be initiated by stalling of RNA polymerase II on a lesion [13, 14] and involves recruitment of the ATP-dependent chromatin remodeling protein CSB and the WD40 domain containing protein CSA [15–17]. In yeast, the CSB ortholog Rad26 [18] is also implicated, but no functional homolog for CSA has been identified. DNA damage that occurs elsewhere in the genome is repaired by global genome NER (GG-NER). Such lesions are recognized in mammals by the UV-DDB ubiquitin ligase complex and the heterotrimeric complex XPC/hHR23/Centrin-2 [19–24]. In yeast, detection of lesions depends also on XPC and hHR23 orthologs Rad4 and Rad23 [25, 26], but it involves a different ubiquitin ligase complex consisting of Rad7, Rad16, Cul3, and Elc1 proteins [27]. Following detection of a lesion, the general transcription factor IIH (TFIIH) is recruited to the site of damage [28, 29]. Using its XPB and XPD helicase subunits, TFIIH locally unwinds a stretch of approximately 30 nucleotides around the lesion, providing access for other repair factors. Other essential DNA-binding proteins XPA and RPA are also recruited and thought to stimulate translocation and damage verification by TFIIH [30] and stabilize and orient the endonucleases which incise DNA around the damage [31]. Next, a stretch of approximately 25–30 nucleotides of DNA surrounding the lesion is excised by the structure specific endonucleases XPF/ERCC1 and XPG [32–34]. Finally, the resulting gap is filled in by DNA synthesis and ligation, involving replication factors such as PCNA and RFC and several ligases and polymerases [35–37].

The strong conservation of NER proteins across different life domains suggests that NER must be an important, universal repair pathway. This is also evident from the severe symptoms that are associated with NER deficiency in mammals [38]. Rare UV-sensitive hereditary disorders such as xeroderma pigmentosum (XP), Cockayne syndrome (CS), and trichothiodystrophy (TTD) are caused by specific mutations in NER genes. XP is characterized by extreme cancer proneness, whereas CS and TTD exhibit segmental progeria and neurodevelopmental problems. In mice, these symptoms are phenocopied by similar mutations [39]. Some mutations in specific NER genes, such as those found in ERCC1 and XPF [40, 41], are associated with severe growth and developmental defects. This shows that even during normal growth, that is, even without excessive exposure to genotoxic agents, the function of these specific NER proteins is essential for normal development and life. 

In spite of its strong evolutionary conservation, NER does not always function in exactly the same manner in different organisms. For instance, TC-NER does not seem to be active in *Drosophila *[42]. Furthermore, the yeast RAD23/RAD4 complex is not only involved in GG-NER, as in mammals, but also in TC-NER [43, 44]. Furthermore, cells from different tissues can respond differently to UV irradiation [45] or modify NER activity such that it is active in transcribed genes only [46]. It is thus likely that in different cells, other NER-regulatory pathways are activated. Although the basic mechanism by which NER removes and repairs damaged DNA is known, it is still not well understood to what level NER or individual NER proteins can be modulated or regulated and how this contributes to differential NER activity in cells. Besides NER, other mechanisms exist that process UV-induced DNA damage. In many organisms, photolyase enzymes directly reverse UV-induced DNA damage following activation by light [47]. This photoreactive repair is, however, not active in placental mammals. In proliferative cells, two emergency strategies can also prevent direct cell killing due to unrepaired UV lesions [48]. First, in S phase, UV lesions cause replication fork collapse and subsequent generation of DNA breaks, which are repaired by HR. Second, damage bypass mechanisms involving specialized translesion polymerases can circumvent damage in S-phase, in an error-prone way. However, to avoid these two unfavorable conditions, break induction, and low fidelity repair, most cells are equipped with efficient DNA damage signaling pathways that activate cell-cycle checkpoints providing more time to properly fix lesions. 

## 3. NER in *C. elegans*

In recent years, the use of the nematode *C. elegans *to study DNA repair pathways has become increasingly intensive. *C. elegans' *main advantages for studying a biological processes such as DNA repair include its relatively fast and easy genetic manipulation, short life cycle, and straightforward recognizable *in vivo *phenotypes. The animal is simply grown on bacteria-seeded culture plates and produces self-fertilized offspring within a few days. Many loss-of-function mutants are available, and its genome, invariable cell lineage, and development are well annotated and accessible via various web resources (http://www.wormbase.org/). Homology searches, protein-protein interaction mapping analysis, and genetic screening have indicated that the major repair pathways found in mammals are conserved to the molecular level in *C. elegans* [6, 7, 49]. These pathways include BER [50–52], NHEJ, HR [5], ICL repair [7], mismatch repair [53, 54], and NER.

Almost 30 years ago the first UV-sensitive *C. elegans *mutants were identified and described [55]. These so-called *rad *(for abnormal radiation sensitivity, see Table 1) mutants were isolated in a screen for animals sensitive to UV or ionizing radiation. In subsequent years, phenotypes of these mutants were extensively characterized [55–61], but to date, the molecular identity of only two *rad *mutants is known. The *rad-5 *locus encodes for the DNA damage checkpoint protein CLK-2 which is an ortholog of yeast Tel2p [62]. The *rad-3 *locus encodes a genuine NER protein, the *C. elegans *XPA ortholog, which is essential for survival following UV irradiation [63]. 

Orthologs of most of the known NER proteins have been identified by homology searches in *C. elegans *(Table 2). RNAi-mediated knockdown and loss-of-function mutations of many NER proteins has confirmed their role in the response to UV irradiation and repair of UV photolesions [63, 65, 68–73]. Importantly, no photolyases or photoreactive repair have been observed in *C. elegans* [58, 74]. Together, these results indicate that NER is fully operational and represents the major and only repair pathway which removes UV-induced DNA damage in *C. elegans*, just as in mammals. As will be discussed below, these studies also demonstrate the different response of different tissues to UV irradiation. Furthermore, NER is found to be differently regulated during development and aging and in cells of different tissues. 

**Figure 2:**
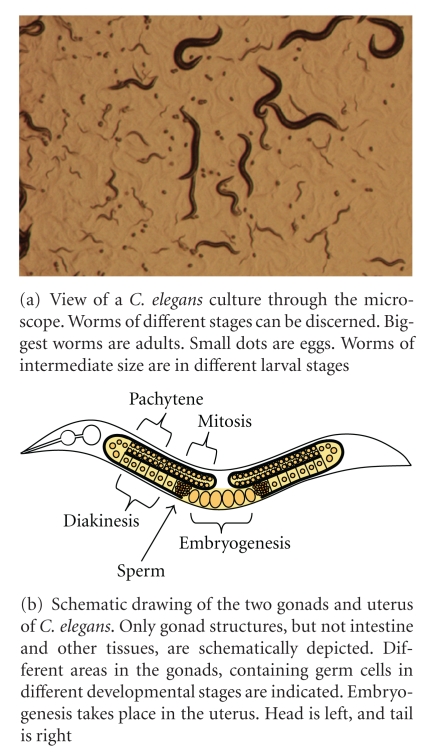
C. elegans.

Because adult *C. elegans *consists of a limited set of 959 somatic cells which still represent many different cell types, the animal seems ideally suited to study the DDR *in vivo *during growth and development of different tissues. Adult *C. elegans *produce approximately 250–300 self-fertilized eggs, which hatch after a few hours. After hatching, larvae develop through four larval stages to become reproducing adults consisting of neuronal, muscular, epithelial, germ line, and other tissue (see Figure 2(a) for a mixed stage *C. elegans *culture). Dauer larvae represent a specialized developmental senescent “survival” stage, in between the L2 and L4 stage. Dauer larvae have been found to be more UV resistant than non-Dauer larvae [63], showing that the response to UV irradiation can change. Increased UV resistance may be a consequence of low levels of transcription in the dauer stage, such that damage does not interfere with this vital process, or because of Dauer-specific upregulation of prosurvival stress response pathways. Understanding how DNA damage leads to different responses in different cells might shed more light on the etiology of symptoms associated with human disease or cancer development.

In mammals, there is a clear distinction between DNA damage recognition via GG-NER and TC-NER, but in other organisms, both subpathways utilize the same proteins for damage recognition [11], or TC-NER may not function at all [42]. In *C. elegans*, UV-induced DNA damage in highly transcribed genes is more rapidly repaired than damage in poorly transcribed genes [65], which is in line with the existence of TC-NER. Furthermore, *C. elegans *expresses orthologs of the GG-NER specific XPC and hHR23B proteins, called XPC-1 and RAD-23, respectively, and an ortholog of the TC-NER specific CSB protein, called CSB-1. Epistatic analysis of mutant *rad-*23/*xpc-1 *and *csb-1 *animals suggests that these proteins act in parallel pathways in *C. elegans* [71]. Therefore, it is likely that also in *C. elegans *two separate DNA damage recognition mechanisms, GG-NER and TC-NER, exist. However, despite this similarity to mammalian NER, *C. elegans *NER probably still functions slightly different as some NER proteins specifically implicated in GG-NER and TC-NER, DDB2 and CSA, have not yet been identified, and thus might not function in *C. elegans*.

## 4. UV Irradiation of *C. elegans*

It is unknown whether UV irradiation really represents a major source of DNA damage for *C. elegans *in its natural habitat [88]. Still, NER is highly conserved and required to survive exposure to UV irradiation. In nature, *C. elegans*, like other organisms not continuously exposed to solar irradiation, might be more likely to encounter genotoxic chemicals in its food and environment which induce DNA alterations that are targets for NER. However, to study NER, UV irradiation is often used as convenient tool to reproducibly and instantaneously induce large amounts of DNA lesions. Effective and reproducible UV irradiation experiments with the worm depend on several conditions. Most studies use UV-C light (254 nm) as damaging agent, which is very potent in generating 64PP and CPD photolesions, because it almost equals the maximum absorbance peak of DNA. An important drawback of UV-C light is its high absorption by water and biopolymers causing a low penetrance of tissue compared to UV light with higher wavelengths. Because *C. elegans *consists of multiple cell layers, we considered the application of a higher wavelength that still produces 64PP and CPD photolesions. Indeed, we have found that UV-B light (302 nm) produces a similar response of *C. elegans *as UV-C light, but it generates better reproducible results [71]. Another means to circumvent absorption problems might be the use of chemicals that induce lesions which are specifically processed by NER. However, such chemicals, like 4-nitroquinoline-1-oxide or N-acetyl-2-aminofluorene [89], have so far not been extensively tested in *C. elegans.*

When irradiating *C. elegans*, care must be taken with respect to shielding effects. For instance, the standard OP50 *E. coli *on which *C. elegans *is cultured [90] forms a relatively thick lawn that partially shields animals from UV light and causes variable results. To avoid this shielding, *C. elegans *should be irradiated in the absence of bacteria. Alternatively, if it is difficult to get rid of bacteria, HT115(DE3) *E. coli *can be used which form a thin lawn. 

## 5. Repair Kinetics in *C. elegans*

It has been noted that late larval stages and adults (Figure 2(a)) are more resistant to UV radiation than younger animals, which can be partially attributed to shielding effects, as they are bigger in size. Shielding may also partially explain why multicellular organisms such as *C. elegans *can tolerate higher doses of UV irradiation than mammalian cells in monolayer culture. Size-related shielding effects are evident from the frequency of lesion induction by UV irradiation [58]. UV-C irradiation produces on average 0.4 to 0.5 lesions per 10 kb per 100 J/m^2^ in young adult *C. elegans* [65]. In smaller animals such as L1 larvae, however, lesion frequency is higher, approximately 4 lesions/10 kb/100 J/m^2^. Two studies have examined kinetics of UV-lesion repair, one using 64PP and CPD antibody-binding radioimmunoassay [58], the other using qPCR on the polymerase epsilon gene to detect polymerase-stalling lesions [65]. Both studies revealed that the global photolesion repair rate in *C. elegans *is comparable to that in cultured human cells but slower than in yeast and bacteria. However, in mammalian cells 64PPs are repaired at a much faster rate than CPDs [91], whereas in *C. elegans*, both photolesions are repaired at the same rate. 

Initial repair rates immediately after UV irradiation seem to remain constant from embryogenesis to early adulthood although at later developmental stages more photoproducts remain unrepaired after 24 hrs [58]. In adulthood, repair seems to be biphasic in the sense that initial repair rates are higher than those after 24 hrs [65]. Furthermore, starting from adulthood, repair rates also decline as the animals age. This is not because global transcription levels or transcription of NER genes diminishes, but it was suggested to be correlated to reduced levels of ATP in aging animals. In general, NER genes are more highly expressed during embryogenesis than during adulthood. However, expression levels during adulthood remain constant and appear sufficient for repair to take place [65, 92]. 

## 6. UV Response of Germ Cells and Embryos

UV irradiation negatively influences germ cell and embryonic development, egg laying and male fertility [56, 71]. *C. elegans *germ cells are contained in two U-shaped gonads, which are joined together at their proximal ends to a common uterus (Figure 2(b); [93]). In the most distal parts of the gonads, germ nuclei mitotically proliferate and migrate in a proximal direction. Upon progression towards the uterus, nuclei further replicate DNA and enter meiosis prophase I. Just before the gonad bend (Figure 2(b)), meiotic nuclei exit pachytene stage, in which homologous chromosomes align and meiotic recombination is initiated and enter diplotene and subsequently diakinesis stage. Half of the germ cells in the pachytene stage are eliminated by apoptosis, probably to maintain tissue homeostasis [94]. As diakinesis stage cells further progress and pass through the spermatheca, they are fertilized, finish meiosis I and II, and initiate first cell divisions of embryogenesis. Because of its transparency and amenable manipulation, the *C. elegans *germ line has become an ideal tool to study DNA repair during the process of meiosis [6].

Progression and maturation of germ cells is blocked by DNA damage. Ionizing and UV radiation, as well as genotoxic chemicals, cause a transient cell cycle arrest of proliferating nuclei and increased apoptosis of pachytene stage nuclei [66]. Furthermore, unrepaired UV damage also blocks further maturation of pachytene cells and/or exit to diplotene [71]. Surprisingly, following UV irradiation, both induction of cell-cycle arrest and apoptosis depend on NER activity [72] and require either the GG-NER or TC-NER pathway [71]. Apoptosis induction furthermore involves the same checkpoint signaling proteins that are also involved after ionizing radiation, such as orthologs of the 9-1-1 signaling complex, the PI_3_ kinases ATM and ATR and p53 [72]. Because UV-induced apoptosis and recruitment of checkpoint proteins also depends on some members of the HR pathway, such as orthologs of MRE11 and RAD54 but not RAD51, it was suggested that processing of a UV-lesion by NER is necessary for HR proteins to activate checkpoint signaling [73]. How this might be accomplished is still not understood, but it is reminiscent of studies in yeast and mammals which also show processing of UV lesions by NER as a prerequisite to activate checkpoint signaling [95, 96]. 

**Figure 3:**
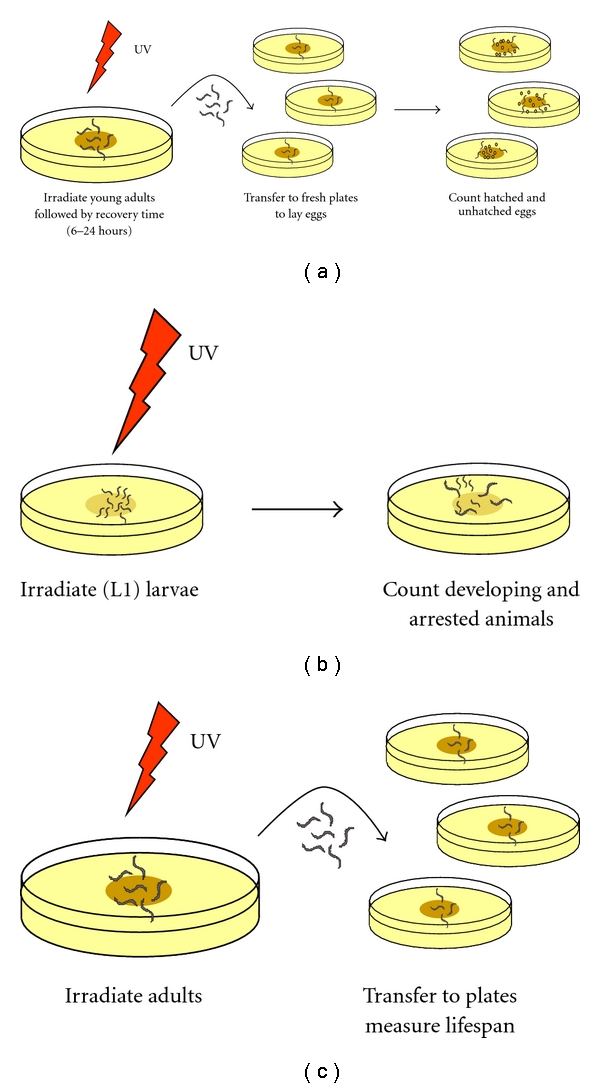
Assays to measure UV sensitivity. Shown are commonly used assays to measure survival of (a) germ cell and embryos, (b) larvae, and (c) adults.

UV irradiation of the germ line causes embryonic lethality, especially if NER is compromised [63, 71, 72]. Embryonic lethality can be used as an easy readout to test whether a protein is involved in NER or the overall UV response (Figure 3(a)). Surprisingly, we found that survival of germ cells, meiotic maturation and repair of lesions after UV in the germ line specifically depends on GG-NER, as inactivation of only this pathway, and not TC-NER, renders germ cells sensitive to UV [71]. Only in a GG-NER deficient background does a TC-NER defect become essential, showing that TC-NER is active but that UV survival mainly depends on GG-NER. The importance of GG-NER in germ cells likely reflects the need of this immortal cell line to protect the integrity of the entire genome.

In contrast to germ cells, growth of *C. elegans *early embryos is relatively resistant to the induction of DNA damage by UV irradiation [55, 97]. Strikingly, a dose of UV which normally induces cell-cycle arrest in mitotic germ cells does not interfere with replication and timing of the cell cycle during the first embryonic cell division. This intriguing damage resistance was attributed to an actively suppressed checkpoint response, which otherwise halts the cell cycle for repair to take place [64]. Checkpoint suppression is dependent on the *rad-2 *gene, one of the originally identified *rad *genes, and the *polh-1 *and *gei-17 *genes. The translesion polymerase POLH-1 is responsible for replicating damaged DNA. It is an ortholog of human POLH, which is mutated in the so-called variant form of the UV-sensitive XP syndrome [98]. In *C. elegans, *its knockdown also renders early embryos and germ cells hypersensitive to UV irradiation [82]. The PIAS1-related E3 SUMO ligase *gei-17 *actively protects POLH-1 from being degraded after damage induction, thereby allowing replication of damaged DNA to occur [99]. 

NER proficient late embryos are even less sensitive to UV irradiation than early embryos [56, 71]. Paradoxically, late *xpa-1 *mutant embryos seem to be more sensitive than early *xpa-1 *embryos [56, 63]. It is not clear why this is, but it might involve differences in checkpoint silencing or transcription dependence.

## 7. Larval Development and Aging

UV irradiation causes proliferating somatic cells in *C. elegans *larvae to arrest. This is visible by growth cessation, but not immediate death, of larvae. Like the embryonic lethality following irradiation of germ cells (Figure 3(a)), this arrest is an easy measure to test involvement of a protein in the UV response or NER (Figure 3(b)). NER deficient larvae are extremely sensitive to UV irradiation and arrest permanently at low doses of UV [63, 71]. Interestingly and in contrast to germ cells, TC-NER is the main repair pathway that is essential for survival and counteracts the growth arrest after UV irradiation [71]. GG-NER seems to be less important and only becomes significant for survival if TC-NER is deficient. Unlike in germ cells, this likely reflects the fact that only actively expressed genes need to be maintained in the mortal somatic cell lineages. 

Permanent developmental arrest induced by UV irradiation does probably not depend on activation of checkpoint signaling, as knockdown of known UV-response checkpoint proteins does not alleviate arrest [63]. However, high UV doses induce permanent transcription block of a reporter gene and degradation of RNA polymerase II (*ama-*1) in *xpa-1 *animals. Therefore, developmental growth arrest is likely due to transcription inhibition. In adult animals, in which somatic cells do not proliferate any more, UV irradiation causes the animals to become smaller, feed less, and live shorter, which is severely increased in NER deficient animals [92]. This reduction of growth could also be caused by lack of transcription. Additionally, it was suggested that UV might inhibit endoreduplication of epidermal syncytium nuclei, which normally increases cell size and drives adult growth in *C. elegans *[100]. 

Besides an effect on growth and development, DNA damage might also affect aging of adult *C. elegans*, as it does in mammals. *C. elegans* is a commonly used model organism to study aging, that lives on average for 2-3 weeks (under lab conditions), in which the insulin/IGF1 pathway regulates lifespan as it does in other organisms [101]. In mammals, there is also strong evidence that aging is in part caused by stochastic accumulation of damage to biomolecules such as DNA, caused by various environmental and metabolic agents [38, 41, 102]. Several human progeroid syndromes are caused by defective DNA repair mechanisms, in particular NER. 

There is some evidence that suggests that longevity and repair are also linked in *C. elegans*. For instance, long-lived mutants are more resistant to oxidative stress and UV irradiation [86, 103–105]. Furthermore, increased UV resistance [105] and NER activity itself [86] were reported to be dependent on the insulin/IGF1 pathway. Still, a clear view on the relation between DNA repair and aging is blurred because of several seemingly contradictory reports. An early examination by Hartman and coworkers of four inbred strains with different life spans found no correlation with DNA repair competence [59]. Furthermore, of all *rad *mutants, only *rad-2* had a severely shortened lifespan [106]. Conflicting data exist on the life span of *xpa-1* mutants. We and others (unpublished data; [63, 92]) find that *xpa-1 *mutants have a similar lifespan as wild type, but others have shown a shorter lifespan for these mutants [86, 87, 106]. These differences may be due to differences in experimental procedures, such as temperature which affects lifespan or the use of FUDR which is applied to prevent egg laying during life span assays. FUDR blocks DNA synthesis causing genomic stress which may, therefore, have an unanticipated effect on lifespan of DNA repair mutants. Also, wild-type life spans differ in each laboratory, which may lead to different conclusions when comparing wild-type and *xpa-1 *lifespans. Furthermore, human patients and mouse models with XPA deficiency do not show progeroid features. Therefore, in addition to *xpa-1, *other NER deficient mutants should be studied to determine whether or not a relationship between NER, DNA damage and aging exists in *C. elegans*. Another unresolved issue is the fact that aging in mammals is a process that takes place in organisms that still have many proliferative tissues and stem cells with the capacity for cell renewal, whereas the somatic tissues of aging *C. elegans *do not proliferate.

Even if NER deficiency by itself might not be sufficient to shorten lifespan of *C. elegans*, UV-mediated induction of DNA damage severely shortens life span. A single or daily low UV dose is extremely limiting to lifespan in *xpa-1 *animals but not in NER proficient animals. A higher dose (e.g., ≥50 J/m^2^ UV-C) also limits wild-type life span but still to a much lesser extent [86, 92]. In addition to a reduced lifespan, UV exposed *xpa-1 *mutants also exhibit other features of aging, such as damaged tissues and internal vacuoles. Thus, a negative correlation between NER deficiency and life span shortening in *C. elegans *might be explained by the lack of sufficient DNA damage accumulation during the short life time of *C. elegans *compared to mammals. If more damage is artificially induced, life span is severely shortened. Like UV-induced embryonic lethality and larval arrest, this UV-induced lifespan reduction is sometimes used as a measure to test involvement of a protein in the UV response or NER (Figure 3(c)).

Whole genome expression profiling of NER-deficient progeroid mice has suggested that in rapid aging tissue growth hormone (insulin/IGF1) signaling is downregulated whereas antistress response pathways are upregulated [38, 41, 102, 107]. As this response is similar to what happens in aging tissue, it was suggested that this reflects a compensatory survival response to counteract aging [38]. Whole genome profiling of *C. elegans *under standard laboratory conditions did not show major differences (>4-fold up- or downregulated genes) between wild type and *xpa-1 *animals [92]. However, using a different methodology and less stringent criteria for up- and downregulation (≥1.8-fold), many transcripts were found to be differentially regulated between wild-type and *xpa-1 *mutants [87]. Gene Ontology enrichment analysis showed a bias of differentially regulated genes belonging to biological pathway clusters such as adult lifespan determination, ER unfolded protein response, regulation of carboxylic acid metabolism and phosphate transport. This enrichment is reminiscent of the suppression of the somatotroph axis and upregulation of stress response pathways in XPA-deficient mouse dermal fibroblast [108], as well as NER-deficient mice [41, 102]. This might mean that some transcriptomic changes associated with NER deficiency and aging might be conserved between *C. elegans *and mammals. Following UV irradiation, several genes and biological networks potentially involved in a stress response were found to be differentially regulated in a similar manner in wild-type and *xpa-1 *animals [92]. Importantly, NER genes as well as most other DNA repair genes are not transcriptionally induced after UV irradiation in *C. elegans*, which is similar to the lack of strong transcriptional regulation in mammals [109] but in contrast to bacteria [110] and yeast [111]. In summary, there is evidence that links NER deficiency and aging in *C. elegans, *but some results are still ambiguous. So far, all studies have made use of the *xpa-1 *mutant which, although it is completely NER deficient, in mice is not strongly associated with accelerated aging. Therefore, studies utilizing other NER-deficient animals may be necessary to deduce whether *C. elegans *can be used as model for the damage accumulation theory of aging. 

## 8. *C. elegans* as Model to Study the UV-Induced DNA Damage Response

In conclusion, studies on different aspects of the UV response confirm the important role of NER in *C. elegans*. Importantly, recent studies using *C. elegans *represent excellent examples of the different DNA damage responses in distinct cell types [64, 71, 72]. These experiments also show that survival and growth following DNA damage are not necessarily linked. This is for instance also evident from the fact that NER-deficient Dauer larvae survive UV irradiation, but are incapable of resuming normal development [63]. Thus, an organism's response to UV irradiation depends very much on its developmental status, its proliferative capacity, and its different tissues and cells.

Many aspects of NER and its role in the UV-DDR are still not well understood. In addition to studying the *in vivo *context of NER in *C. elegans, *this organism is also well suited to genetically identify new NER or UV-DDR regulatory pathways. Although it is not expected that novel core NER genes will be identified, genetic screening of *C. elegans *might prove useful to better understand the context of proteins and pathways in which NER plays a role and by which NER is regulated. In a first attempt to identify such proteins, we have recently identified several ATP-dependent chromatin remodeling factors that are essential for an efficient UV response [71]. Further characterization of novel genes and the cellular responses to UV in *C. elegans *will undoubtedly help to better understand the function of this important DNA repair pathway and etiologies of DNA damage-associated diseases.

## Figures and Tables

**Table 1:** List of original *rad *mutants.

Locus	Gene	Sensitivity	Other affected processes	Repair	References
*rad-1*		UV, IR		Normal	[55, 56, 58]
*rad-2*		UV, IR, MMS	Embryonic checkpoint	Normal	[55, 56, 58, 64]
*rad-3*	*xpa-1*	UV, MMS	Germ line apoptosis	Absent	[55, 56, 58, 63, 65]
*rad-4*		UV, MMS	X chromosome nondisjunction		[55]
*rad-5*	*clk-2*	UV, IR	Checkpoint, longevity		[55, 62, 66]
*rad-6*		UV, IR	Embryogenesis		[55]
*rad-7*		UV, IR	Embryogenesis	Normal	[55, 56, 58]
*rad-8*		UV, oxygen	Embryogenesis, longevity		[55, 67]
*rad-9*		UV	Embryogenesis		[55]

**Table 2:** Nucleotide excision repair genes in *C. elegans. *

Mammalian gene		*C. elegans* gene	Available alleles	Sensitivity to UV	Additional affected processes	References
CETN2		*R08D7.5?*	*tm3611*			
DDB1		*ddb-1*	*tm1769*		Development, protein turnover	[75]
ERCC1		*ercc-1*	*tm1943*			
			*tm1981*			
			*tm2073*	yes		[71]
ERCC4 (XPF)		*xpf-1*	*e1487*	yes	Meiosis	[71, 76–79]
			*tm2842*	yes*		
			*ok3039*	no*		
ERCC5 (XPG)		*xpg-1*	*tm1670*	yes	Apoptosis	[71, 73]
			*tm1682*	yes	Apoptosis	[71, 73]
ERCC6 (CSB)		*csb-1*	*ok2335*	yes		[71]
LIG1		*lig-1*	RNAi		Growth, development	
LIG3		*K07C5.3*				
PCNA		*pcn-1*	*ok1905*		Growth, development	
			*tm3157*			
			*tm3241*		Growth, development	
POLD1		*F10C2.4*			embryogenesis	[80, 81]
POLH		*polh-1*	RNAi		DNA repair	[64, 79, 82]
POLK		*polk-1*	RNAi		Growth, embryogenesis, DNA repair	[79–81]
RAD23B		*rad-23*	*ok1910*			
			*tm2595*	yes		[71]
			*tm3690*			
RFC	RFC1	*rfc-1*	RNAi		Mutator, embryogenesis	[81, 83]
	RFC2	*rfc-2*	RNAi		Embryogenesis	[81]
	RFC3	*rfc-3*	RNAi		Mutator, embryogenesis	[81, 83]
	RFC4	*rfc-4*	RNAi		Growth, development	[80, 81]
	RFC5	*F44B9.8*	RNAI		Embryogenesis	
RPA	RPA1	*rpa-1*	RNAi		Embryogenesis	[80]
	RPA2	*rpa-2*	*ok1627*		Growth, development	
TFIIH	CCNH	*cyh-3*	RNAi		Growth, development	[80]
	CDK7	*cdk-7*	*ax224*		Transcription, cell cycle	[84]
	GTF2H1	*R02D3.3*	RNAi		Growth, development	[80]
	GTF2H2	*T16H12.4*	*tm1767*			
			*tm4960*			
	GTF2H3	*Zk1128.4*	*ok1200*		Growth, development	
			*tm1501*		Growth, development	
	GTF2H4	*Y73F8A.24*	RNAi		Embryogenesis	[81]
	GTF2H5	*Y55B1AL.2*				
	MNAT1	*mnat-1*	*tm2959*		Development	
	ERCC3 (XPB)	*Y66D12A.15*	RNAi		Apoptosis, embryogenesis	[73, 81, 85]
	ERCC2 (XPD)	*Y50D7A.2*			Apoptosis, embryogenesis	[73, 81, 85]
XPA		*xpa-1*	*mn157*	yes		[55, 63]
			*ok698*	yes	Apoptosis, lifespan?	[63, 65, 71, 86, 87]
			*gk674*			
XPC		*xpc-1*	*ok734*	no*	Apoptosis	[72]
			*tm3886*	yes	Apoptosis	[71, 72]

Some phenotypes were taken from http://www.wormbase.org/ (WS221). For those genes for which no alleles are known, results from RNAi experiments are indicated.

*Represent unpublished results.
